# Distinctive Core Histone Post-Translational Modification Patterns in *Arabidopsis thaliana*

**DOI:** 10.1371/journal.pone.0001210

**Published:** 2007-11-21

**Authors:** Kangling Zhang, Vaniyambadi V. Sridhar, Jianhua Zhu, Avnish Kapoor, Jian-Kang Zhu

**Affiliations:** 1 Mass Spectrometry Facility, School of Medicine, Loma Linda University, Loma Linda, California, United States of America; 2 Department of Botany and Plant Sciences, University of California at Riverside, Riverside, California, United States of America; Temasek Life Sciences Laboratory, Singapore

## Abstract

Post-translational modifications of histones play crucial roles in the genetic and epigenetic regulation of gene expression from chromatin. Studies in mammals and yeast have found conserved modifications at some residues of histones as well as non-conserved modifications at some other sites. Although plants have been excellent systems to study epigenetic regulation, and histone modifications are known to play critical roles, the histone modification sites and patterns in plants are poorly defined. In the present study we have used mass spectrometry in combination with high performance liquid chromatography (HPLC) separation and phospho-peptide enrichment to identify histone modification sites in the reference plant, *Arabidopsis thaliana*. We found not only modifications at many sites that are conserved in mammalian and yeast cells, but also modifications at many sites that are unique to plants. These unique modifications include H4 K20 acetylation (in contrast to H4 K20 methylation in non-plant systems), H2B K6, K11, K27 and K32 acetylation, S15 phosphorylation and K143 ubiquitination, and H2A K144 acetylation and S129, S141 and S145 phosphorylation, and H2A.X S138 phosphorylation. In addition, we found that lysine 79 of H3 which is highly conserved and modified by methylation and plays important roles in telomeric silencing in non-plant systems, is not modified in Arabidopsis. These results suggest distinctive histone modification patterns in plants and provide an invaluable foundation for future studies on histone modifications in plants.

## Introduction

Core histones, in the form of an octamer consisting of two copies of H2A, H2B, H3 and H4, are wrapped by 147 bp DNA to form the nucleosome [1]. Multiple nucleosome units are lined up to form beads-on-a-string structure, which can be further compacted into a 30 nm fiber-the foundation of chromatin structure, through the assistance of linker histone H1 and the binding of transcription factors or cofactors [2]. Chromatin can be either extended to form an open, active euchromatin accessible to transcription factors/cofactors or condensed to form a closed, silent heterochromatin inaccessible to transcription factors/cofactors [3]. Histone modifications play a vital role in determining these states of chromatin during growth and development [4].

Histone post-translational modifications take place mainly on the N-terminal tails of histones. Histone methylation together with other modifications (acetylation, phosphorylation, ubiquitination, sumoylation and ADP-ribosylation) orchestrates an important functional role in gene expression and a “histone code” was accordingly hypothesized [5]. The modifications such as acetylation and methylation of lysine residues, are conserved at some lysine residues of histones while not at others. For example, lysine 5, 8, 12 and 16 of histone H4 are acetylated in many species including humans, fly and yeast. However, the modifications at some other lysines, for example, H3 lysine 9 and lysine 27 can be both acetylated and methylated in humans but only acetylated in budding yeast and only methylated in chicken erythrocytes [6]–[8]. Histones can also be modified outside their N-terminal regions, as evidenced by the recent discovery of methylation of lysine 79 and acetylation of lysine 56 of histone H3 via mass spectrometry analysis, which is supported by subsequent genetic studies [7], [9]–[11].

Mass spectrometry is capable of not only providing direct information on the site and type of modification, and differentiating between two nominally isobaric modifications (i.e., acetylation versus tri-methylation), but also implementing quantitative analysis. For example mass spectrometry can be used to determine the acetylation level at certain lysine residues and in some cases, also the level of methylation at specific lysines (such as lysine 4 and lysine 79 of H3) [7], [12]–[13]. Mass spectrometry thus has been widely recognized as an irreplaceable tool for studying histone modifications and in combination with chromatin immunoprecipitation (ChIP) and/or immunofluorescence assays has the potential for identifying new histone modification sites, modification patterns, and for genome-wide chromatin-related functional studies [14]–[15].

The reference plant, *Arabidopsis thaliana*, is an excellent model organism for studies on epigenetic regulation. Histone modification patterns have been shown to be critical for establishing and maintaining stable epigenetic states of genes in *Arabidopsis*. For example, prolonged cold treatment (i.e. vernalization) triggers increases in H3K9 and H3K27 dimethylation and decreases in H3K4 trimethylation and histone acetylation at the FLC locus, causing a stable repression of FLC that is maintained through mitosis even at warm temperatures [16]–[17]. The Polycomb Repressive Complex 2-mediated repression of FLC is necessary for flowering of the *Arabidopsis* plants [18]. *Arabidopsis* is also an outstanding model system to dissect the interplay between small interfering RNAs (siRNAs), DNA methylation and histone modifications [19]–[23]. In plants, 24 nt siRNAs can direct DNA methylation of complementary sequences, causing stable transcriptional gene silencing [19]. The heterochromatic marker, H3K9 methylation, can direct DNA methylation by the plant specific CMT3 DNA methyltransferase [24]–[25]. On the other hand, DNA methylation is also required for high level H3K9 methylation [26]–[27]. Notwithstanding the importance of histone modifications in chromatin regulation in plants, only the N-terminal modifications on *Arabidopsis* histone H3 have been systematically analyzed by mass spectrometry combined with chromatographic separation [28]. The analysis found several conserved modifications on histone H3 such as methylation at K4, K9, K27 and K36 and acetylation at K14 and K18 [28]. We have used mass spectrometry to analyze modifications of all core histones in *Arabidopsis.* Our results not only confirm acetylation and methylation at some conserved lysine residues in the four core histones (H2A, H2B, H3 and H4), but also reveal many unexpected distinctive modifications at other sites [29]. These unique modifications include acetylation at K20 of H4, acetylation at K6, K11, K27 and K32, phosphorylation at S15 and ubiquitination at K143 of H2B, acetylation at K144 and phosphorylation at S129, S141 and S145 of H2A, and phosphorylation at S138 of H2AX. In addition, we did not find any modification at lysine 79 of H3 which is highly conserved in non-plant systems. Collectively, our analysis of core histone modification sites here, albeit still incomplete, will be invaluable for future studies of histone modifications in plant genetic and epigenetic regulation.

## Results

### Identification of modification sites in H2A

The histone H2A fraction collected from HPLC eluant was digested by trypsin and analyzed by liquid chromatography-tandem mass spectrometry (LC/MS/MS) on a QTOF instrument. The acquired raw data were converted to peak-list (PKL) files, which were submitted for MASCOT searching of proteins and protein post-translational modification sites. The TIC trace of the signature immonium ion of acetylated lysines at *m/z* 126.1 was also displayed to monitor the ion intensity for manually analyzing the MS/MS spectra of acetylated peptides, which could be missed by software search.

**Figure 1:**
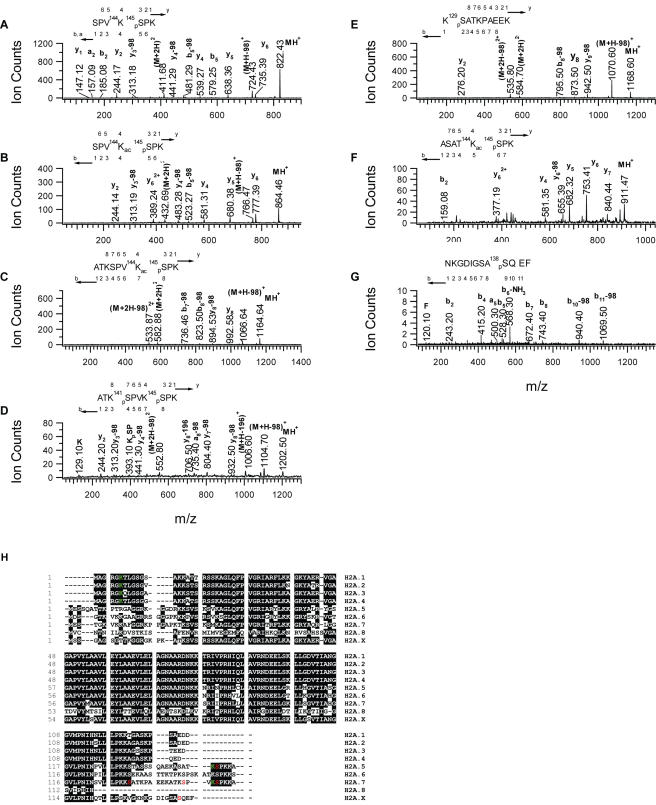
Identification of H2A modification sites. A. MS/MS spectrum of doubly-charged precursor ion at *m/z* 411.72 demonstrating phosphorylation at S145 in the peptide SPVK^145^_p_SPK of H2A.7. B. MS/MS spectrum of doubly-charged precursor ion at *m/z* 432.69 demonstrating phosphorylation at S145 and acetylation at K144 in peptide SPV^144^K_ac_^145^_p_SPK of H2A.7. C. MS/MS spectrum of doubly-charged precursor ion at *m/z* 582.88 demonstrating phosphorylation at S145 and acetylation at K144 in peptide ATKSPV^144^K_ac_^145^_p_SPK of H2A.7. D. MS/MS spectrum of doubly-charged precursor ion at *m/z* 601.78 demonstrating phosphorylation at S141 and S145 in peptide ATK^141^_p_SPVK^145^_p_SPK of H2A.7. E. MS/MS spectrum of the doubly-charged precursor ion at *m/z* 584.80 demonstrating phosphorylation at S129 in peptide K^129^_p_SATKPAEEK of H2A.7. F. MS/MS spectrum of the doubly-charged precursor ion at *m/z* 456.24 demonstrating phosphorylation at S145 and acetylation at K144 in the peptide ASAT^144^K_ac_^145^_p_SPK of H2A.5. G. MS/MS spectrum of the doubly-charged precursor ion at *m/z* 666.81 demonstrating phosphorylation at S138 in the peptide NKGDIGSA^138^_p_SQEF of H2AX. H. Sequence alignment of H2A isoforms (H2A.1-H2A.8). K in green color: acetylation; S in red color: phosphorylation.

In *Arabidopsis thaliana*, H2A has eight isoforms (H2A.1–H2A.8) (Fig. 1H). Among the isoforms, H2A.1, H2A.2, H2A.3 and H2A.4 share a similar N-terminal sequence AGRGKT(Q)TLGSGS(V, A , G)AK where serine at position 11 of H2A.1 is replaced by either valine (H2A.2) or glycine (H2A.4), or two residues threonine and serine at position 6 and 11 are replaced by glutamine and alanine (H2A.3). We found that the lysine 5 in all the four H2A isoforms was acetylated (Fig S2). For instance, from the fragmentation ions of the four H2A peptides with sequences shown in Figure S2A–D, an observation of the immonium ion at m/z 126 and a 42 mass unit added to lysine 5 indicated an acetylation rather than tri-methylation at lysine 5. The fragmentation ions, namely “b” and “y” ions labeled in individual figures (Figure S2A–D), matched well with the peptide sequences of four H2As given by the NCBI accession number gi 15223708 (H2A.1), 15232330 (H2A.2), 15237024 (H2A.3) and 15239697 (H2A.4). Histone H2A is known to be conservatively acetylated at lysine 5 and lysine 9 in other species including human, bovine, chicken erythrocyte and yeast (yeast equivalent sites K4 and K7) [7], [29]. In the plant H2A and its isoforms, there is no lysine at position 9 or its vicinity for acetylation (Table 1 and Fig. 1H).

**Table 1:** Comparison between plant and non-plant H2A modifications

Modification Sites			Modification Types			Function
A. thaliana	H. sapiens	S. cerevisiae	A. thaliana	H. sapiens	S. cerevisiae	
**K**5 (H2A.1-H2A.4)	**K**5	**K**4	Acetylation	Acetylation	Acetylation	Transcriptional Activation
	**K**9	**K**7		Acetylation	Acetylation	Transcriptional Activation
**K**144 (H2A.5, H2A.7)			Acetylation			
	**S**1	**S**1		Phosphorylation	Phosphorylation	Crb2 recruitments
**S**129 (H2A.7)	**T**120	**S**121	Phosphorylation	Phosphorylation	Phosphorylation	Cell cycle progression (Mitosis)
**S**141 (H2A.7)			Phosphorylation			
**S**145 (H2A.5, H2A.7)			Phosphorylation			
**S**138 (H2A.X)	**S**139 (H2A.X)		Phosphorylation	Phosphorylation		DSB repair
**K**128 (H2A.5-H2A.7)	**K**119	**K**120	Not detected	Ubiquitination	Not detected	Hox gene silencing

Next, we determined whether there were other modifications (mainly phosphorylation and ubiquitination) associated with histone 2A. Particular attention was given to the known modification sites observed in other species. S1 is a known H2A phosphorylation site observed in human and yeast. However, in *Arabidopsis* H2A, S1 is replaced by alanine in isoforms H2A.1-H2A.4 and by other amino acids (E, D or V) in isoforms H2A.5-H2A.8. Therefore, S1 phosphorylation cannot exist in *Arabidopsis*. Ubiquitination at lysine 120/119 of H2A was observed in human/*Drosophila* with the consensus peptide sequence (P)K^119/118^KT [30]–[31], while K128, similar to K120 in yeast, in the same consensus sequence in *Arabidopsis* (and yeast) was found to be free of ubiquitination (Table 1). Surprisingly, in the H2A isoform H2A.7 (gi 15238549) a marked amount of phosphorylation was detected at serine 145 (estimated by the ion intensity) in the C-terminal extension even without enrichment for phospho-peptides (Fig. 1A). Considering the sulphuric acid used for the precipitation of histones from nucleosomes and that the process might decompose most of the phospho-histones, we used cation exchange (using Bio-Rex 70) column chromatography to purify DNA binding proteins including histones followed by trypsin digestion and enrichment of phospho-peptides by a mixture of anion exchange resin and TiO_2_. LC/MS/MS analysis of this “treated” sample detected six additional phospho-peptides. They are H2A.7 peptide SPV^144^K_ac_^145^_p_SPK (MS/MS spectrum shown in Fig. 1B) where K144 is acetylated and S145 is phosphorylated, peptide ATKSPV^144^K_ac_^145^_p_SPK (MS/MS spectrum shown in Fig. 1C) where K144 is acetylated and S145 is phosphorylated, peptide ATK^141^_p_SPVK^145^_p_SPK (MS/MS spectrum shown in Fig. 1D) where S141 and S145 are both phosphorylated, peptide K^129^_p_SATKPAEEK (MS/MS spectrum shown in Fig. 1E) where S129 is phosphorylated, and H2A.5 peptide ASAT^144^K_ac_^145^_p_SPK (gi 15241016) (MS/MS spectrum shown in Fig. 1F) where K144 is acetylated and S145 is phosphorylated. The acetylation of lysine (K144) next to the phosphorylated serine (S145) in H2A.5 and H2A.7 was unexpected, and the observation provides evidence of coexistence of acetylation and phosphorylation at two adjacent residues (K and S). Because of the similarity of C-terminal sequence of H2A.6 (gi 15241857) to those of H2A.5 and H2A.7, K144 and S145 (Fig. 1F) in H2A.6 are likely acetylated and phosphorylated, although they were not detected by mass spectrometry. Unlike K5 acetylation observed on the N-terminus of H2A.1-H2A.4, we did not detect acetylation on the N-terminus of H2A.5-H2A.7, possibly because acetylation enzymes (histone acetyltransferases) like phosphorylation enzymes (kinases) also recognize specific peptide consensus sequence which is absent in these three H2A isoforms (Figure 1H) [32]. More interestingly, we were able to identify S138 phosphorylation of H2A variant H2A.X (gi 15221875) from the MS/MS fragmentation of the doubly-charged precursor ion at *m/z* 666.90 (Fig. 1G). It is also noteworthy that we did not find any modifications in H2A.8 (gi 15236314), possibly because either H2A.8 has non-conservative N- and C-terminal sequences or the protein/its modification level was too low to be detected by mass spectrometry.

### Identification of modification sites in H2B

**Figure 2:**
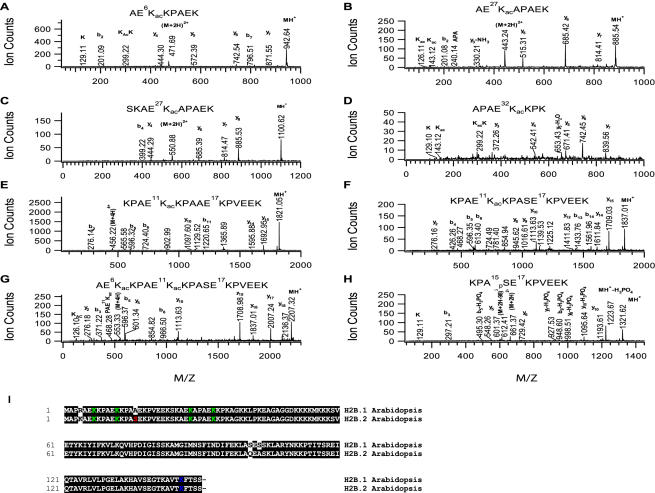
Identification of H2B modification sites. A. MS/MS spectrum of the doubly-charged precursor ion at *m/z* 471.69 showing H2B acetylation site at K6 in the peptide AE^6^K_ac_KPAEK. B. MS/MS spectrum of the doubly-charged precursor ion at *m/z* 443.24 showing H2B acetylation site at K27 in the peptide AE^27^K_ac_APAEK. C. MS/MS spectrum of the doubly-charged precursor ion at *m/z* 550.88 for determining H2B acetylation site at K27 in the peptide SKAE^27^K_ac_APAEK. D. MS/MS spectrum of the triply-charged precursor ion at *m/z* 304.15 for determining H2B acetylation site at K32 in the peptide APAE^32^K_ac_KPK. E. MS/MS spectrum of the quadruply-charged precursor ion at *m/z* 456.01 for determining H2B.1 acetylation site at K11 in the peptide KPAE^11^K_ac_KPAAEKPVEEK. F. MS/MS spectrum of the quadruply-charged precursor ion at *m/z* 460.01 for determining H2B.2 acetylation site at K11 in the peptide KPAE^11^K_ac_KPASEKPVEEK. G. MS/MS spectrum of the quadruply-charged precursor ion at *m/z* 552.70 for determining H2B.2 acetylation sites at K6 and K11 in the peptide AE^6^K_ac_KPAE^11^K_ac_KPASEKPVEEK. H. MS/MS spectrum of the doubly-charged precursor ion at *m/z* 661.37 for determining H2B.2 phosphorylation site in the peptide KPA^14^_p_SEKPVEEK. I. Sequence alignment of *Arabidopsis* H2B (H2B.1 and H2B.2). K in green color: acetylation; S in red color: phosphorylation.

The HPLC fraction containing H2B was digested by trypsin and analyzed by LC/MS/MS. From the MS/MS spectrum of the precursor ion at *m/z* 471.8 (Figure 2A), a peptide with the sequence AE^6^K_ac_KPAEK from the N-terminus of H2B was obtained where lysine 6 was determined to be acetylated. This was based on the detection of y6 and y7 ions corresponding to the fragmentation of an acetylated peptide rather than the neutral loss of y6-59 and y7-59 ions corresponding to the fragmentation of a tri-methylated peptide. Similar analyses can be applied to the peptide AE^27^K_ac_APAEK with doubly-charged precursor ion at *m/z* 443.24 where K27 was determined to be acetylated by MS/MS (Fig. 2B); the peptide SKAE^27^K_ac_APAEK with doubly-charged precursor ion at *m/z* 550.88 where K27 was determined to be acetylated by MS/MS (Fig. 2C); the peptide APAE^32^K_ac_KPK with triple-charged precursor ion at *m/z* 304.17 where K32 was determined to be acetylated by MS/MS (Fig. 2D); the H2B.1-specific peptide KPAE^11^K_ac_ KPAAE^17^KPVEEK with quadruple-charged precursor ion at *m/z* 456.01 where K11 was determined to be acetylated by MS/MS (Fig. 2E); the peptide KPAE^11^K_ac_ KPASE^17^KPVEEK specific to H2B.2 with quadruple-charged precursor ion at *m/z* 460.01 where K11 was determined to be acetylated by MS/MS (Fig. 2F); and the peptide AE^6^K_ac_KPAE^11^K_ac_ KPASEKPVEEK specific to H2B.2 with quadruple-charged precursor ion at *m/z* 552.70 where both K6 and K11 were determined to be acetylated by MS/MS (Fig. 2G). Therefore, our mass spectrometric analysis revealed four acetylation sites on the N-terminus of *Arabidopsis* H2B (Fig. 2I), compared with six acetylation sites on the N-terminus of human [33] and yeast H2B (Table 2 and Fig. 2I).

**Table 2:** Comparison between plant H2B modifications and non-plant H2B modifications

Modification Sites			Modification Types			Function
A. thaliana	H. sapiens	S. cerevisiae	A. thaliana	H. sapiens	S. cerevisiae	
		**K**3 (H2B.1)			Acetylation	
**K**6	**K**5	**K**6	Acetylation	Acetylation	Acetylation	
**K**11	**K**11		Acetylation	Acetylation		
	**K**12	**K**11		Acetylation	Acetylation	
	**K**15	**K**16		Acetylation	Acetylation	
	**K**16	**K**21		Acetylation	Acetylation	
**K**27		**K**22	Acetylation		Acetylation	
**K**32	**K**23		Acetylation	Acetylation		
**S**15 (H2B.2)	**S**14	**S**10	Phosphorylation	Phosphorylation	Phosphorylation	Apoptosis
	**S**32			Phosphorylation		Apoptosis
**K**143	**K**120	**K**123	Ubiquitination	Ubiquitination	Ubiquitination	Transcriptional activation

Using MASCOT search we were able to determine the ubiquitin protein (Score 138 and 4 peptide sequences matched) in the HPLC fraction containing H2B protein, indicating that H2B was likely ubiquitinated. To confirm this, we loaded the H2B fraction on a SDS-PAGE gel and found in addition to the H2B band the presence of a band (approximate 27 kDa) larger in size than the major H2B band (∼19 kDa). We further confirmed that this larger band corresponded to ubiquitinated form of H2B by Western-blot analysis with ubiquitin antibody [34]. Based on previous results that histone H2B is mono- ubiquitinated at lysine 123 on the C-terminal peptide with sequence AVT^120^KYTSS in human or AVT^123^KYSSS in yeast, we speculated that lysine 143 of plant H2B in the C-terminal sequence AVT^143^KFTSS may be ubiquitinated. Since trypsin cuts after arginine in the C-terminus of ubiquitin, it leaves two glycine residues of the ubiquitin, resulting in an increment of 114 dalton to the peptide mass. This increase in mass can be used for the detection of ubiquitination site [35]–[36]. We manually examined the MS/MS spectrum of the precursor ion at *m/z* 477.7 (doubly-charged and 57 Da added to the mass of the peptide AVTKFTSS) whose fragmentation ions were analyzed by Prospector_Product (prospector.ucsf.edu) . Indeed, the MS/MS fragmentation ions matched well the peptide sequence AVT_GG_KFTSS with GG chain attached to the lysine after trypsin digestion, confirming that H2B lysine 143 is ubiquitinated in plants [34]. It is of interest to note that the ubiquitinated H2B peptides in human, yeast and plant all had an aromatic amino acid residue, either tyrosine (Y) or phenylalanine (F), next to the ubiquitinated lysine residue (Fig. 2I).

Next, we searched for H2B phosphorylation sites. As illustrated for H2A phosphorylation site identification, LC/MS/MS analysis of the ‘treated’ sample by cation exchange (using Bio-Rex 70 resin) column chromatographic purification of histones and the phospho-peptide enrichment protocol identified phosphorylation at S15 in H2B.2. As shown in Fig. 2H, MS/MS fragmentation pattern of the doubly-charged precursor ion at *m/z* 661.37 matched the peptide sequence KPA^15^_p_SEKPVEEK where S15 is phosphorylated. This assignment of phosphorylation was supported by the observation of the ion at *m/z* 1223.67 (converted from the doubly-charged ion at *m/z* 612.38) corresponding to the neutral loss of H_3_PO_4 _from the peptide ion at *m/z* 1321.62 (converted from the doubly-charged ion at *m/z* 661.37) and the observation of ‘y’ and ‘b’ satellite ions that are 98 (loss of H_3_PO_4_) dalton smaller than their corresponding y and b ions.

### Identification of modification sites of H3

The HPLC fraction containing H3 was analyzed in two parts; one portion was digested by trypsin and analyzed by LC/MS/MS directly, and the other was digested by Arg_C and separated further into sub-fractions by HPLC before analysis by matrix assisted laser desorption/ionization-time of flight (MALDI-TOF) mass spectrometry. From LC/MS/MS runs of the trypsin digests, we examined thoroughly all the modification sites already known in other species, by both MASCOT search and manual analyses of the MS/MS raw data. As shown in Figure S3A, from the MS/MS spectrum of the precursor ion at *m/z* 472.32, the fragmentation ions (the majority is y ions) established the peptide sequence KSTGG^14^K_ac_APR in which acetylation at lysine 14 was assigned. This assignment is based on the fact that a functional group with 42 Da nominal mass was added to lysine 14 and that the fragmentation pattern was consistent with the one previously observed for the same lysine 14-acetylated peptide in other species [7]. Similarly, Figure S3B illustrates the fragmentation of the precursor ion at *m/z* 493.3, which was consistent with peptide ^9^K_ac_STGG^14^K_ac_APR where both lysine 9 and 14 are acetylated. We also observed four precursor ions containing modification information for lysine 18 and 23. Precursor ion at *m/z* 365.72 (doubly charged) corresponded to the peptide ^18^K_ac_QLATK where lysine 18 is acetylated (Figure S3C); precursor ion at *m/z* 450.75 (doubly charged) corresponded to peptide QLAT^23^K_ac_AA R where lysine 23 is acetylated (Figure S3D); precursor ion at *m/z* 514.8 (doubly-charged) corresponded to peptide KQLAT^23^K_ac_AAR where lysine 23 is acetylated (Figure S3E); and precursor ion at m/z 535.8 corresponded to peptide ^18^K_ac_QLAT^23^K_ac_AAR where both lysine 18 and 23 are acetylated (Figure S3F). We note that lysine 56, known to be acetylated in *Drosophila* and yeast [10]–[11], was not found to be acetylated in our *Arabidopsis* samples.

**Figure 3:**
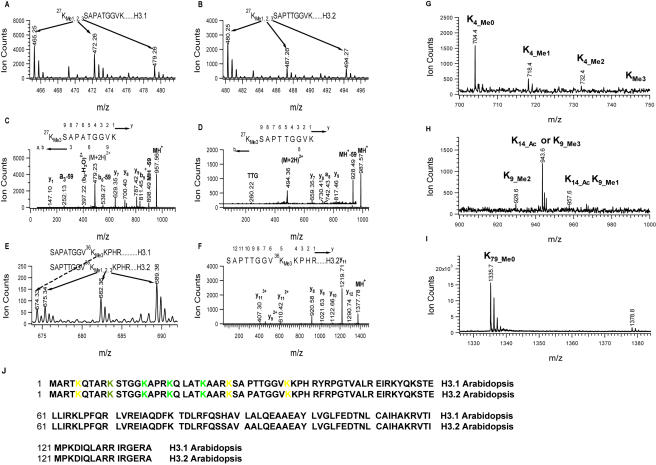
Identification of H3 methylation sites. A. ESI mass spectrum showing mono- (*m/z* 465.25), di- (*m/z* 472.26) and tri-methylation (*m/z* 479.26) at K27 of H3.1. B. ESI mass spectrum showing mono- (*m/z* 480.25), di- (*m/z* 487.26) and tri-methylation (*m/z* 494.27) at K27 of H3.2. C. MS/MS spectrum of the doubly-charged precursor ion at *m/z* 479.26 for determining tri-methylation at K27 of H3.1. D. MS/MS spectrum of the doubly-charged precursor ion at *m/z* 494.27 for determining tri-methylation at K27 of H3.2. E. ESI-MS spectrum showing mono- (*m/z* 675.34), di- (*m/z* 682.36) and tri-methylation (*m/z* 689.36) at K36 in H3.2 and tri-methylation (*m/z* 674.33) at K36 of H3.1. F. MS/MS spectrum of the doubly-charged precursor ion at *m/z* 689.36 for determining tri-methylation at K36 of H3.2. G. MALDI-TOF mass spectrum showing non- (*m/z* 704.4) mono- (*m/z* 718.4), di- (*m/z* 732.4) and tri-methylation (746.4) at K4. H. MALDI-TOF mass spectrum showing di-methylation (*m/z* 929.6) at K9, acetylation at K14 or tri-methylation (*m/z* 943.6) at K9, acetylation at K14 and mono-methylation (*m/z* 957.6) at K9. I. MALDI-TOF mass spectrum showing no methylation at K79. J. Sequence alignment of H3 isoforms (H3.1 and H3.2). K in green color: acetylation; K in yellow color: methylation; K in olive color: both acetylation and methylation. Note: All peptides in A–F were released from trypsin digestion and peptides in G–I were released from Arg-C digestion.

We next examined all of the potential methylation sites of histone H3. Three groups of ions, at *m/z* 465.25, 472.26, 479.26 (Fig. 3A), at *m/z* 480.25, 487.26, 494.27 (Fig. 3B), and at *m/z* 675.34, 682.36, 689.36 (Fig. 3E) were detected to be the precursor ions of potentially methylated peptides since a mass difference of 7 units (doubly-charged) between two nearby peaks was observed. Interestingly, the first group of ions matched with the nominal mass of the peptide ^9^KSTGG^14^KAPR where lysine 9 could be mono- to tri-methylated (K9_Di-methylation at *m/z* 465.3; K9_Tri-methylation at *m/z* 472.3; K9_Mono-methylaiton & K14_Acetylation at *m/z* 479.3). The MS/MS spectra of those three ions could not establish the sequence of KSTGGKAPR, instead the spectra corresponded to the sequence K_me1-3_SAPATGGVK of H3 isoform H3.1 where lysine 27 is mono-, di- and tri-methylated with mono-methylation being the dominant form (Figure 3A). The production ion spectrum of the precursor ion at *m/z* 479.26 was shown here to illustrate lysine 27 tri-methylation (Figure 3C). The observation of MH^+^-59, a_3_-59 and b_6_-59 ions produced from the neutral loss of tri-methyl amine confirmed the presence of tri-methylation rather than acetylation at lysine 27.

Similarly, the second group of ions (at *m/z* 480.25, 487.26 and 494.27) were unambiguously assigned to the peptide ^27^K_me1-3_SAPTTGGVK of H3 isoform H3.2 which has one amino acid residue different from H3.1, that is at position 31 with threonine replacing alanine. MS/MS fragmentations again supported the assignment of mono-, di- and tri-methylation at lysine 27. The fragmentation pattern of the tri-methylated peptide with the precursor ion at *m/z* 494.27 was shown in Figure 3D. The clearly observed MH^+^-59 ion supported the presence of tri-methylation at lysine 27 in the peptide sequence ^27^K_me3_SAPTTGGVK. From the ESI-MS spectra of the third group ions, we concluded that lysine 36 in H3.2 is mono-, di- and tri-methylated, with tri-methylation being the most dominant form. The peak to the left of the peak at *m/z* 674.33 corresponded to the above stated lysine 36 mono-methylated peptide of H3.2 and matched the mono-isotopic mass of peptide SAPATGGV^36^K_me3_KPHR of H3.1, suggesting that lysine 36 of H3.1 is also tri-methylated. There was no detection of mono- and di-methylated ions (the mass region is not shown), possibly because they were too weak to be detected. Thus, our mass spectrometric data show that lysine 27 and lysine 36 in plants are mono-, di- and tri-methylated, and lysine 27 is prone to being more mono-methylated while lysine 36 is more tri-methylated. Our results demonstrate that K27 methylation and K36 methylation could each exist exclusively without the other as we have observed here or co-exist as previously reported [28].

From the LC/MS/MS runs, we did not detect methylation at lysine 79. We could not obtain clear information on lysine 4 modification because the tryptic peptide containing K4 was short, hydrophilic and was eluted together with the solvent front (using formic acid as the ion-pairing agent). We also could not determine K9 methylation during the LC/MS/MS runs possibly because the majority of K9 was acetylated. So, we ran an HPLC using 0.1% Trifluoroacetic acid (TFA) as the ion-paring agent to separate the peptides from the Arg_C digest of H3. Arg_C protease prefers to cut arginine, leaving modified and un-modified lysine immune to cleavage. Each fraction of LC was collected and analyzed by MALDI-TOF mass spectrometry. In our LC system, H3 Arg_C digested peptides containing K4, K9 and K79 had relative retention time of 10, 15 and 35 minutes, respectively. We found that lysine 4 is largely mono-methylated, with detectable but significantly lower amounts of di- and tri-methylation (Figure 3G). For lysine 9, we found di-methylation (Figure 3H) based on the fact that the ion at *m/z* 929.60 is eluted in an earlier fraction than those lysine 27-containing peptides [7]. Again, for lysine 79 we did not detect any methylation by MALDI. A very strong single peak of un-methylated peptide with mono-isotopic mass at *m/z* 1335.7 was detected while the peaks corresponding to the mono-methylated form (at *m/z* 1349.7), di-methylated form (at *m/z* 1363.7) and tri-methylated form (at *m/z* 1377.7) were missing (Figure 3I). A peak observed at *m/z* 1378.8 corresponding to a different peptide was labeled to distinguish it from the tri-methylated K79 peak. Table 3 and Fig. 3J summarize the H3 modification sites in human, yeast and *Arabidopsis*.

**Table 3:** Comparison between plant H3 modifications and non-plant H3 modifications

Modification Sites			Modification Types			Function
A. thaliana	H. sapiens	S. cerevisiae	A. thaliana	H. sapiens	S. cerevisiae	
**K**4	**K**4	**K**4	Methylation	Methylation	Methylation	Transcriptional activation
**K**9	**K**9		Methylation	Methylation		Transcriptional repression
**K**9	**K**9	**K**9	Acetylation	Acetylation	Acetylation	Transcriptional activation
**K**14	**K**14	**K**14	Acetylation	Acetylation	Acetylation	Transcriptional activation
**K**18	**K**18	**K**18	Acetylation	Acetylation	Acetylation	Transcriptional activation
**K**23	**K**23	**K**23	Acetylation	Acetylation	Acetylation	Transcriptional activation
**K**27	**K**27		Methylation	Methylation		Transcriptional repression
	**K**27	**K**27		Acetylation	Acetylation	
**K**36	**K**36	**K**36	Methylation	Methylation	Methylation	Transcriptional repression/activation
**K56**					Acetylation	DNA replication
**K**79	**K**79	**K**79	Not detected	Methylation	Methylation	Telomere silencing
**S**10	**S**10	**S**10	?	Phosphorylation	Phosphorylation	Mitotic condensation
**S**28	**S**28	**S**28	?	Phosphorylation	Phosphorylation	Mitotic condensation

### Identification of modification sites of H4

From a single LC/MS/MS run of the tryptic digests of histone H4, we obtained a total of seven acetylated peptides. As shown in Fig. S4A–F, the MS/MS spectra indicate that lysine 16 is acetylated in the peptide GGA^16^K_ac_R with isotopic mass at *m/z* 265.67 (doubly-charged) (Fig. S4A); lysine 12 is acetylated in the peptide GLG^12^K_ac_GGAK with isotopic mass at *m/z* 365.2 (deconvoluted singly-charged ion at *m/z* 729.44) (Fig. S4B); lysine 8 is acetylated in the peptide GG^8^K_ac_GLG with mono-isotopic mass at *m/z* 329.7 (deconvoluted singly-charged ion at *m/z* 658.40) (Fig. S4C); two lysines K5 and K8 are acetylated in the peptide G^5^K_ac_GG^8^K_ac_GLGK with mono-isotopic mass at *m/z* 443.2 (deconvoluted singly-charged ion at *m/z* 885.55) (Fig. S4D); two lysines K12 and K16 are acetylated in the peptide GLG^12^K_ac_GGA^16^K_ac_R with mono-isotopic mass at *m/z* 464.3 (deconvoluted singly-charged ion at *m/z* 927.56) (Fig. S4E); and three lysines K8, K12, K16 are acetylated in the peptide GG^8^K_ac_GLG^12^K_ac_GGA^16^K_ac_R with mono-isotopic mass at *m/z* 606.33 (Fig. S4F). The seventh potentially acetylated peptide with mono-isotopic mass at *m/z* 279.2 (doubly-charged) was established by the MS/MS fragmentation pattern as KILR where the lysine residue is modified with a group, either acetyl or tri-methyl, whose unit mass is 42 dalton (Fig. S5). For the ion at m/z 126.1, a signature for acetylated lysines, was observed in the MS/MS spectrum (Fig. S5D) indicating the presence of acetylation and not tri-methylation for the modified lysine [7], [12]. HPLC was also run to isolate modified peptides from the trypsin digest of H4 and the peptides were subsequently analyzed by MALDI-TOF (Fig. S5A–C). We note that the modified peptide was eluted after the un-modified peptide (see peak 7 & 8 in Fig. S5C), demonstrating that the former was more hydrophobic than the latter. Except for the two ions related to the un-modified and modified KILR peptides whose masses differed by 42 Da, we did not observe mono- (addition of 14 Da) or di- (addition of 28 Da) methylated peptides. Taken together, we conclude that the lysine residue in KILR was acetylated. Histone H4 contains a tryptic peptide ^20^KILR and lysine 20 is therefore the potential acetylation site.

**Figure 4:**
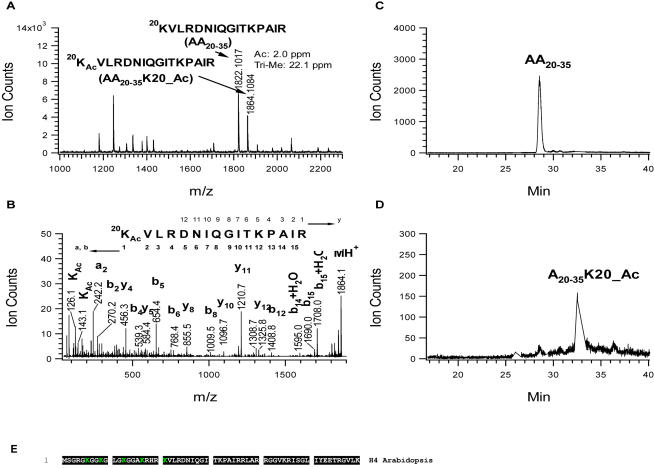
Identification of H4 acetylation sites at K20. A. MALDI-TOF mass spectrum of HPLC fraction of Arg_C digest containing K20 peptide. B. MALD-MS/MS spectrum of the precursor ion at *m/z* 1864.14 for determining acetylation at K20. C. Chromatogram of the ion at *m/z* 1822.10 (doubly-charged ion at *m/z* 911.55). D. Chromatogram of the ion at *m/z* 1864.11 (doubly-charged ion at *m/z* 932.55). E. Sequence alignment of *Arabidopsis* H4. K in green color: acetylation.

Because the peptide K_ac_VLR was short, we could not completely rule out the possibility that other proteins co-eluted with H4 from HPLC separation had the same tryptic peptide. We therefore performed an Arg_C digestion of H4 for the purpose of obtaining a peptide with an extended sequence of KILR by controlling the digestion efficacy. Indeed, we were able to isolate two peptides, the un-modified peptide ^20^KVLRDNIQGITKPAIR and the modified counterpart which eluted at around 40 minute from the HPLC column using TFA as the ion-paring agent. Mono-isotopic masses of the two peaks differed by 42.067 using MALDI-TOF mass spectrometric measurement (Fig. 4A). Using the mass of the un-modified peptide (known peptide sequence and confirmed by ESI-MS/MS) to calibrate the MALDI spectrum, we found that the modified peptide gave a mono-isotopic mass 1864.1084 with only 2.0 ppm error for the assignment of acetylation as compared to 22.1 ppm for the assignment of tri-methylation (Fig. 4A). Furthermore, a MALDI-TOF collision induced dissociation (CID) experiment was done towards the modified peptide with mono-isotopic mass at m/z 1864.1. The fragmentation ions, namely “a”, “b” and “y” ions, established the peptide sequence ^20^K_ac_VLRDNIQGITKPAIR where lysine 20 was acetylated while lysine 31 was not (Fig. 4B). The observation of signature immonium ions at m/z 126.1 and 143.1 for acetylated lysine gave additional support for acetylation. The pool of peptides was re-injected on the LC column and LC/MS/MS analysis was run using formic acid as the ion-pairing agent. Besides obtaining sequence information of the two peptides with un-acetylated and acetylated lysine 20 (data not shown), we also noticed that the modified peptide was eluted about 4 minutes after the un-modified one (Fig. 4C & 4D), which is in agreement with the notion of acetylation increasing the hydrophobicity of a molecule [37]. Collectively these evidences support that lysine 20 of plant histone H4 is acetylated. Table 4 and Fig. 4E summarize the H4 modification sites in human, yeast and *Arabidopsis*.

**Table 4:** Comparison between plant H4 modifications and non-plant H4 modifications

Modification Sites			Modification Types			Function
A. thaliana	H. sapiens	S. cerevisiae	A. thaliana	H. sapiens	S. cerevisiae	
	**S**1	**S**1		phosphorylation	phosphorylation	DSB re-joining
**K**5	**K**5	**K**5	Acetylation	Acetylation	Acetylation	Transcriptional repression
**K**8	**K**8	**K**8	Acetylation	Acetylation	Acetylation	Transcriptional activation
**K**12	**K**12	**K**12	Acetylation	Acetylation	Acetylation	Transcriptional activation
**K**16	**K**16	**K**16	Acetylation	Acetylation	Acetylation	Transcriptional activation
	**K**20	**K**20		Methylation		Heterochromatin silencing
**K**20			Acetylation			

## Discussion

We used a combination of various mass spectrometric methods including LC/MS/MS, MALDI-TOF and MALDI-CID as well as HPLC purification to identify histone modification sites in *Arabidopsis thaliana.* We also successfully implemented a phospho-peptide enrichment method to identify histone phosphorylation sites. Our results show that plant histones are extensively acetylated and/or methylated at most lysine sites that are also acetylated and/or methylated in mammals and yeast. Histone arginine methylation was not detected, possibly because of its low abundance or because it blocks cleavage by trypsin. The following modifications were found: acetylation at K5 and K144 of histone H2A; acetylation at K6, K11, K27 and K32 of histone H2B; acetylation at K9, K14, K18, K23 of histone H3; acetylation at K5, K8, K12, K16 and K20 of histone H4; methylation at K4, K9, K27 and K36 of H3; phosphorylation at S145 of H2A.5, at S129, S141 and S145 of H2A.7, at S138 of H2AX, and at S15 of H2B; ubiquitination at K143 of H2B. However, several modifications conserved in non-plant systems were missing or different in plants. For example, we found no methylation at lysine 79 of H3 and no methylation but acetylation at lysine 20 of H4.

Four *Arabidopsis* H2A isoforms H2A1-H2A.4 are acetylated at K5, a conserved H2A acetylation site in human and other species, while other three *Arabidopsis* H2A isoforms H2A.5-H2A.7 are acetylated at the C-terminal K144 and phosphorylated at S145. To our knowledge, these two modifications (acetylation at K144 and phosphorylation at S145) have not been reported previously to co-exist. There were no indications of modifications on H2A.8 whose N-terminal and C-terminal sequences are dissimilar to other H2A isoforms. The diversity of modification pattern in *Arabidopsis* H2A and the exclusive acetylation/phosphorylation preference of isoforms either on the N-terminus or on the C-terminus add additional complexity to the unique modification patterns of *Arabidopsis*. Moreover, we report for the first time the identification by mass spectrometry the DNA damage marker-H2A.X phosphorylation site, S138 of *Arabidopsis* H2A.X. The phosphorylation of an equivalent residue in human H2A.X, S139, has been well studied [38].

H2B was determined to be acetylated at four sites (K6, K11, K27 and K32) on the N-terminus. We observed that the yeast acetylated peptide consensus AEK_ac_K (A) also applies to plants, implicating the existence of plant homologs of the yeast H2B acetyltransferase(s). We also noticed that lysine before proline could hardly be acetylated (i. e. K7, K12, K17, K33), in contrast to the fact that lysine after proline can be acetylated as we and others observed acetylation at K11 (after P10) of human H2B. The preference of acetylation at lysine after proline rather than acetylation at lysine before proline most likely arises from the conformational preference or hindrance of proline to an acetyltransferase [39]. S15 was identified as the phosphorylation site of *Arabidopsis* H2B. S15 of *Arabidopsis* H2B is equivalent to S10 of yeast H2B because they are within similar peptide sequence contexts AEKKPASE (E is replaced by K in yeast). While S10 phosphorylation of yeast H2B has been implicated in apoptosis regulation [40], the function of S15 phosphorylation of *Arabidopsis* H2B remains to be determined. We also found that plant H2B was ubiquitinated at lysine 143, equivalent to lysine 123 in yeast H2B and lysine 120 in human H2B. H2B ubiquitination in *Arabidopsis* has also been detected using immunoblot analysis in several recent studies [34], [41], [42]. It is interesting to note that human, yeast and plant H2B share the same peptide sequence AVTKY (F) with the feature that either Y or F is located next to the ubiquitinated lysine residue. The aromatic amino acids Y and F were thought to form base stacking interactions with DNA [43]. In this context, ubiquitination of the lysine next to the aromatic amino acids might disrupt this interaction, suggesting that H2B ubiquitination might be involved in disrupting DNA binding [34].

H3 K79 is mono-, di- and tri-methylated in many mammalian and non-mammalian cell lines and in yeast studied to date. The DOT1 enzyme, an H3 K79 methyltransferase, is responsible for methylation at this site, which has been suggested to play a role in telomeric silencing [9]. Interestingly, in our study, H3 lysine 79 in *Arabidopsis* was found free of modification/methylation. Furthermore, we could not find a DOT1 homolog in *Arabidopsis* by BLAST search using human or yeast DOT1 as the query sequence. It would be of great interest to explore the functional implication of the lack of methylation at lysine 79 in plants. In our study, we also found that K27 from both H3.1 and H3.2 were mono-, di, or tri-methylated, with the dominance of mono-methylation and in the absence of K36 methylation. Likewise, K36 from H3.2 were mono-, di-, tri-methylated with the dominance of tri-methylation and in the absence of K27 methylation, although methylation of K27 and methylation of K36 could coexist in plants [28] and in chicken erythrocytes [7]. Interestingly, the study of Johnson et al [28] showed that dimethylated K36 is higher than mono- or trimethylated forms for H3.2. This discrepancy with our result could be caused by the different plant tissues and ecotypes used. No mono- or di-methylation at K36 was detected on H3.1, possibly because the signals were too low compared with tri-methylation which was the dominant form observed for H3.2.

Lysine 20 of histone H4 was reported to be modified in the form of mono-, di- and tri-methylation in almost all multicellular organisms [44], whereas lysine 20 of budding yeast has not been reported to be modified (acetylation or methylation). To date, at least five methyl transferases have been identified that specifically methylate lysine 20: SET7, SET8, SET9 and Suv4-20h1 and Suv4-20h2, with potential roles ranging from cell cycle regulation, development, gene silencing associated with pericentrimeric heterochromatin to the coordination of transportation of Crb2 to the sites of DNA damage [44]–[47]. However, our results show that lysine 20 of H4 in *Arabidopsis* is free of methylation. Interestingly, our mass spectrometry data revealed the presence of acetylation at lysine 20 of plant H4, supporting the prediction made by Waterborg [48] more than a decade ago. Waterborg predicted acetylation at this site in alfalfa using a radioactive chemical acetylation method to protect the five un-modified lysine residues of the N-terminal 23 amino acids of H4 and counted the radioactivity by steps of hydrolysis [49]. In contrast to the tri-methylation of H4 lysine 20, which is thought to be associated with repressive chromatin, we speculate that acetylation at lysine 20 alone or together with the nearby lysine 16 acetylation may play a role in activating transcription.

In summary, we have used mass spectrometry to identify plant histone modification sites. Although it is still incomplete, the identification of modification sites here sets a a valuable foundation for further studies aimed at a comprehensive identification of histone modifications and their role in plant growth and development.

### Experimental protocol

#### Isolation of core histones from *Arabidopsis thaliana*

*Arabidopsis* plants were grown in soil in a growth chamber for 4 weeks at 22°C with 16 hours of light and 8 hours of dark each day. Around 30 grams of above ground plant tissues were used for histone purification according to the protocol of Waterborg et al [49]. Briefly, nuclei were extracted in isolation buffer [0.25M sucrose, 25 mM HEPES (pH 7.5), 3 mM CaCl_2_, 10 mM NaCl, 1 mM PMSF,1 mM DTT, 0.25% Nonidet 40, leupeptin, and pepstatin A], and deacetylase and phosphatase inhibitors (1 mM sodium butyrate, 1 mM NaF, 1 mM sodium orthovandate and 10 µM calyculin) were added. The nuclei were pelleted by centrifugation at 3000 rpm for 10 min and then washed twice with the isolation buffer. Nuclei were suspended in 5%GuHCl (guanidine hydrochloride in 100mM potassium phosphate buffer (pH 6.8) and passed through Bio-Rex 70 (BIORAD) resin. The bound histones were eluted with 20% GuHCl and dialyzed with water. (Fig S1B)

#### HPLC separation of histones

Core histones were separated into sub-histones in the order of H2B, H4, H2A and H3 by reverse-phase HPLC as described previously [8]. Briefly, a 90-minute of gradient from 38% mobile phase B (0.1% TFA in acetonitrile), 62% mobile phase A (0.1% TFA in water) to 90% B through the time of 55 minutes with 55% mobile phase B, was run on an HP 1100 capillary HPLC instrument (Hewlett-Packard, Palo Alto) using a 150×2.0 mm Phenomenex C4 column running in the normal pump mode at a flow rate of 50 µl/min. (Fig S1A)

#### HPLC purification of peptide from H3 Arg_C digests

Isolated histone H3 was digested by Arg_C in 25 mM ammonium biocarbonate overnight. The digests were SpeedVac dried, re-dissolved in 0.1% TFA and then purified by reverse-phase HPLC using an Agilent 150×0.5 mm (5 µm) Zorbax C18 column performed on the same HP 1100 capillary HPLC running in the micro pump mode. A 100 minute gradient was run from 2% mobile phase B (0.1% TFA in acetonitrile) and 98% mobile phase A (0.1% TFA in water) to 90% mobile phase B through the time of 65 minutes with 65% mobile phase B at a flow-rate of 6 µl/min. A diode array detector (DAD) was used to record the chromatogram and each fraction was manually collected in a 0.5 mL silicanized Eppentorf tube and then dried. The same gradient and column was used for HPLC linked to the mass spectrometry except 0.1% formic acid rather than TFA was used in mobile phases A and B.

#### Phospho-peptide enrichment

Phospho-peptide enrichment was processed as described previously [50]–[51] with minor modifications. Briefly, a mixture of equal amount of TiO_2_ and anion-exchange resin (LC-NH2, Supelco) was placed on the top of C18 ZipTip pipette tips (Millipore) followed by several steps of washing and elution.

#### Electrospray mass spectrometry

Electrospray mass spectrometry was performed on a Waters hybrid quadrupole-time of flight (Q-TOF) mass spectrometer (Waters, Manchester, UK). The Q-TOF was run in a survey mode as described previously [13]. The raw data were converted into peak-list (PKL) files that were processed by MASCOT (www.matrixscience.com) for searching proteins and protein posttranslational modifications sites. *De-novo* sequencing, through the aide of Prospector Product software (prospector.ucsf.edu), was also performed for either confirmation of modification sites reported by MASCOT or to find other modification sites which MASCOT might fail to determine.

#### MALDI-TOF mass spectrometry

MALDI-TOF measurement of peptides from protein digests was performed on the Voyager DE-STR Biospectrometry Workstation (ABI, Foster City, CA) with delayed extraction operated in the reflectron mode using α-cyano-4-hydroxycinnamic acid as the matrix. A MALDI MS/MS experiment was carried out on the QSTAR instrument (ABI, Foster City, CA) equipped with a MALDI source.

## Supporting Information

Figure S1 Separation of Arabidopsis histones. A. HPLC chromatogram of Arabidopsis histones. B. SDS-PAGE of total histones and HPLC fractions.
(0.15 MB PDF)

Figure S2 Identification of H2A (in isoforms H2A.1-H2A.4) acetylation site at K5. A. MS/MS spectrum of the doubly-charged precursor ion at m/z 479.8. B. MS/MS spectrum of the doubly-charged precursor ion at m/z 480.3. C. MS/MS spectrum of the doubly-charged precursor ion at m/z 474.3. D. MS/MS spectrum of the doubly-charged precursor ion at m/z 459.3.
(0.78 MB EPS)

Figure S3 Identification of H3 acetylation sites at K9, K14, K18 and K23. A. MS/MS spectrum of the doubly-charged precursor ion at m/z 472.27 for determining acetylation site at K14. B. MS/MS spectrum of the doubly-charged precursor ion at m/z 493.27 for determining acetylation sites at K9 and K14. C. MS/MS spectrum of the doubly-charged precursor ion at m/z 450.75 for determining acetylation site at K23. D. MS/MS spectrum of the doubly-charged precursor ion at m/z 530.8 for determining acetylation sites at K18 and K23. E. MS/MS spectrum of the doubly-charged precursor ion at m/z 465.7 for determining acetylation site at K18. F. MS/MS spectrum of the doubly-charged precursor ion at m/z 514.8 for determining acetylation site at K23.
(0.99 MB EPS)

Figure S4 Identification of H4 acetylation sites at K5, K8, K12 and K16. A. MS/MS spectrum of the doubly-charged precursor ion at m/z 265.67 for determining acetylation site at K16. B. MS/MS spectrum of the doubly-charged precursor ion at m/z 365.22 for determining acetylation site at K12. C. MS/MS spectrum of the doubly-charged precursor ion at m/z 329.70 for determining acetylation site at K8. D. MS/MS spectrum of the doubly-charged precursor ion at m/z 443.28 for determining acetylation sites at K5 and K8. E. MS/MS spectrum of the doubly-charged precursor ion at m/z 464.28 for determining acetylation sites at K12 and K16. F. MS/MS spectrum of the doubly-charged precursor ion at m/z 606.33 for determining acetylation sites at K8, K12 and K16.
(1.03 MB EPS)

Figure S5 Identification of acetylation at K20 of H4. A. MALDI-TOF mass spectrum of HPLC fraction containing un-modified K20 peptide 20KVLR. B. MALDI-TOF mass spectrum of HPLC fraction containing acetylated K20 peptide 20KacVLR. C. HPLC chromatogram of the Arg_C digested peptides from mixture of H3 and H4. D. MS/MS spectrum of the precursor ion at m/z 279.2 (doubly-charged).
(1.32 MB EPS)
