# Supplementary figures and images for: Distinctive Core Histone Post-Translational Modification Patterns in Arabidopsis thaliana
Source: PLoS One. 2007 Nov 21;2(11):e1210. doi: 10.1371/journal.pone.0001210 (PMC2075165; doi:10.1371/journal.pone.0001210)

Fig. S1  
A

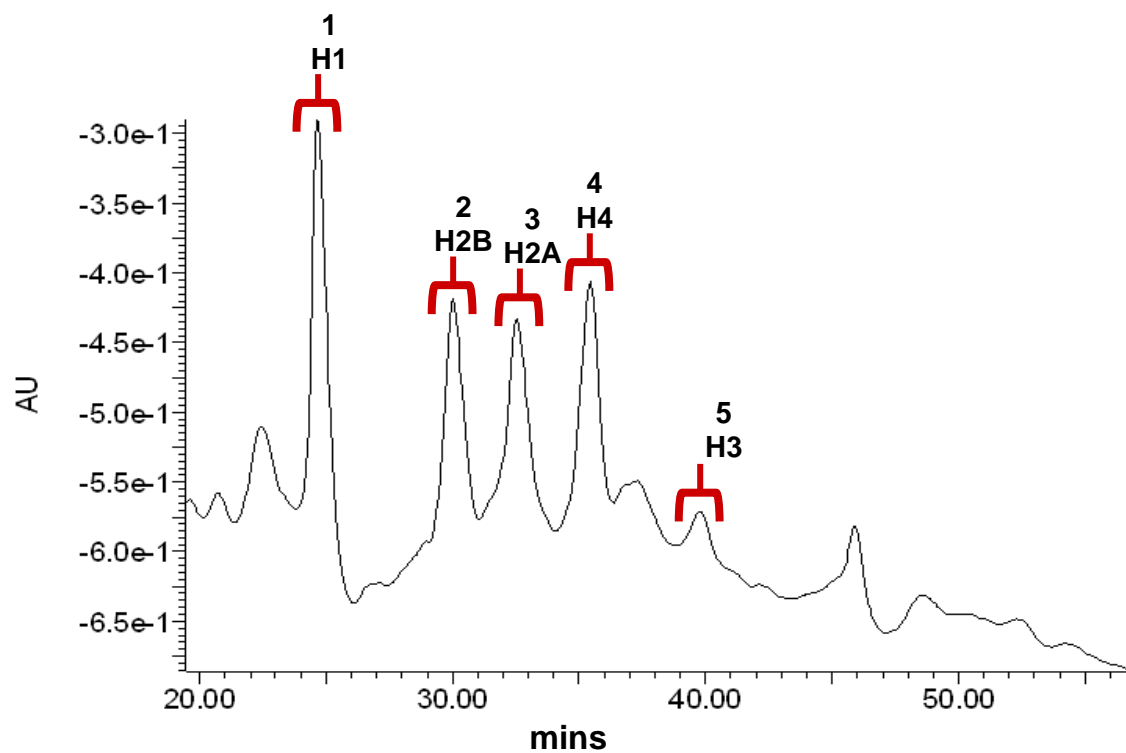

B

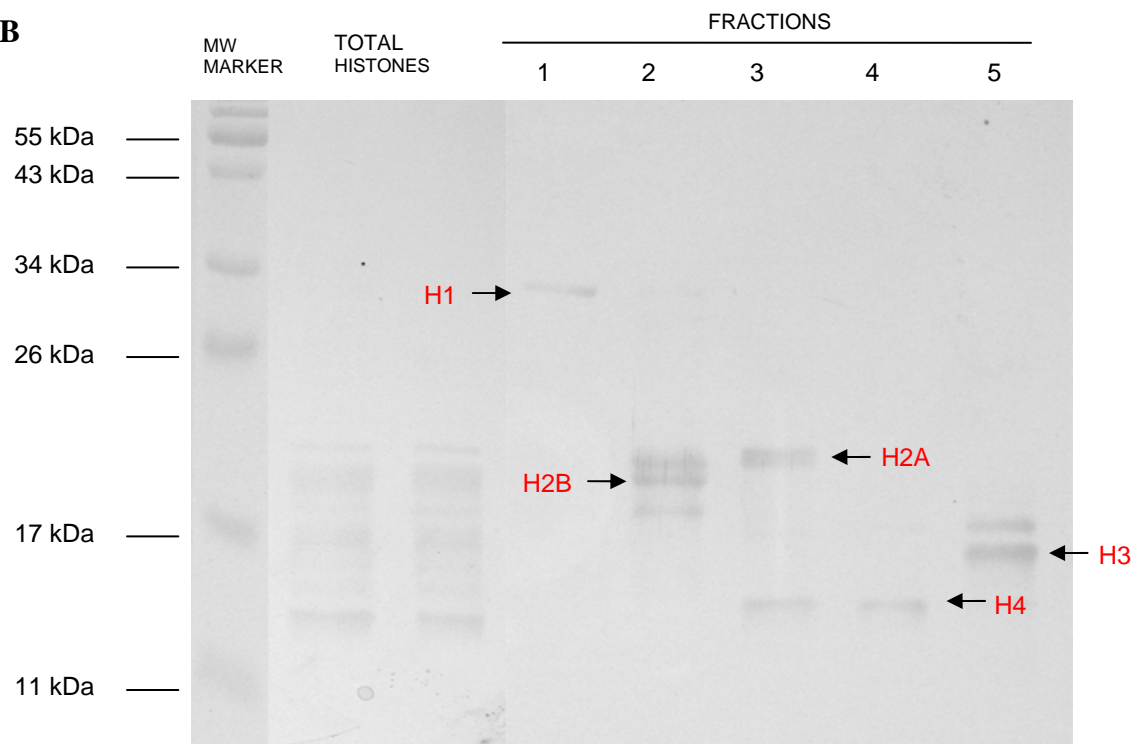

Supplement: Figure S1 — Separation of Arabidopsis histones. A. HPLC chromatogram of Arabidopsis histones. B. SDS-PAGE of total histones and HPLC fractions. (0.15 MB PDF) [file pone.0001210.s001.pdf]
